# Assessing Microbial Corrosion Risk on Offshore Crude Oil Production Topsides under Conditions of Nitrate and Nitrite Treatment for Souring

**DOI:** 10.3390/microorganisms10050932

**Published:** 2022-04-29

**Authors:** Danika Nicoletti, Mohita Sharma, Lisa M. Gieg

**Affiliations:** Department of Biological Sciences, University of Calgary, 2500 University Drive NW, Calgary, AB T2N 1N4, Canada; danika.nicoletti1@ucalgary.ca (D.N.); mohita.sharma@ucalgary.ca (M.S.)

**Keywords:** microbiologically influenced corrosion, MIC, offshore produced water, FPSO, topside, nitrate, nitrite, souring, sulfide

## Abstract

Oilfield souring is a detrimental effect caused by sulfate-reducing microorganisms that reduce sulfate to sulfide during their respiration process. Nitrate or nitrite can be used to mitigate souring, but may also impart a corrosion risk. Produced fluids sampled from the topside infrastructure of two floating, production, storage, and offloading (FPSO) vessels (Platform A and Platform B) were assessed for microbial corrosion under nitrate and nitrite breakthrough conditions using microcosm tests incubated at 54 °C. Microbial community compositions on each individual FPSO were similar, while those between the two FPSO vessels differed. Platform B microbial communities responded as expected to nitrate breakthrough conditions, where nitrate-reducing activity was enhanced and sulfate reduction was inhibited. In contrast, nitrate treatments of Platform A microbial communities were not as effective in preventing sulfide production. Nitrite breakthrough conditions had the strongest sulfate reduction inhibition in samples from both platforms, but exhibited the highest pitting density. Live experimental replicates with no nitrate or nitrite additive yielded the highest general corrosion rates in the study (up to 0.48 mm/year), while nitrate- or nitrite-treated fluids revealed general corrosion rates that are considered low or moderate (<0.12 mm/year). Overall, the results of this study provide a description of nitrogen- and sulfur-based microbial activities under thermophilic conditions, and their risk for MIC that can occur along fluid processing lines on FPSO topsides that process fluids during offshore oil production operations.

## 1. Introduction

Crude oil recovered from offshore reservoirs accounts for approximately 30% of the global crude oil inventory [1]. The offshore oil recovery industry routinely contends with infrastructure corrosion partly due to the use of seawater for secondary recovery source water. While the seawater used is usually treated before injection to prevent debris from clogging the pipeline network [2], many chemical properties of the seawater remain unchanged. As such, approximately 25–30 mmol per L of sulfate is typically present in the injection water, which can stimulate the activity of sulfate-reducing microorganisms (SRMs) that produce sulfide as the end product of their respiration [3]. For close to a century, SRMs have been known as key actors in oilfield souring and microbiologically influenced corrosion (MIC) of oil and gas infrastructures [4,5,6]. Biological souring not only devalues recovered hydrocarbon products due to the requirement of additional refining steps before its downstream use, but also introduces health risks to workers [7,8]. Within oil and gas systems, many SRMs can utilize different external electron donors, such as hydrogen, hydrocarbons, or other organic molecules, to drive sulfide production. In addition, some types of SRMs can use electrons directly from the metallic infrastructure itself to drive sulfide production [3,6]. Such biological sulfide production further yields products known to be highly corrosive to iron infrastructure, such as iron sulfides [6].

Beyond the presence of sulfate, seawater can contain taxa of many different metabolic groups that may also participate in MIC by various mechanisms. For example, nitrate-reducing microorganisms (NRMs) have been implicated in MIC by producing potentially corrosive nitrite [9,10]. Additionally, some NRMs can utilize sulfide as an electron donor (e.g., nitrate-reducing sulfide-oxidizing microorganisms, or NR-SOMs), re-oxidizing it back to sulfate, which can then be used by SRMs as an electron acceptor to continue sulfidogenesis [9,10,11]. The incomplete oxidation of sulfide may also result in the accumulation of corrosive elemental sulfur [12] and/or nitrite, depending on the proportions of sulfide to nitrate in a given system [13]. There are many other types of microorganisms that can participate in MIC, including acetogens, iron-reducers, and methanogens [14,15,16,17,18], and several of these microbial groups have been detected in corroded offshore fluids or infrastructure [2,19,20,21].

Nitrate or nitrite are sometimes used in injection water for souring mitigation [7,8,9,10,11]. The addition of nitrate encourages the growth of NRMs and simultaneously stifles the growth of SRMs by a variety of mechanisms [7,8,22]. The selective growth of NRMs due to their energetically favorable metabolism upon nitrate addition results in the biocompetitive exclusion of SRMs, as NRMs preferentially utilize carbon sources and other trace nutrients. Nitrite, present by injection or as a byproduct of NRM metabolism, inhibits dissimilatory sulfite reductase in the sulfate reduction pathway, preventing the production of sulfide by SRMs [7,8,13,22]. Recent laboratory studies showed that the temperature, in particular, can strongly influence nitrite accumulation when nitrate is used as a souring treatment [23,24]. The studies showed that nitrite accumulated at temperatures >50 °C, effectively inhibiting sulfide production [23,24]. If present, NR-SOMs capable of using sulfide as an electron donor can also work to remove sulfide from the system by re-oxidizing it to sulfate, a less chemically corrosive sulfur species.

The use of nitrate and nitrite as means of souring mitigation has shown varying degrees of success, in practice [24,25,26]. One caveat to the use of nitrate or nitrite as a souring treatment is the risk that each potentially poses to the corrosion of carbon steel infrastructures [9,10,13]. For example, nitrate and nitrite treatments were shown to present general corrosion rates of up to 0.112 mm/year and 0.100 mm/year, respectively, in a laboratory bioreactor study [10]. Experiments using a pure NR-SOM strain (*Sulfurimonas* sp. strain CVO) also showed corrosion rates of up to 0.27 mm/year, when nitrate and sulfide concentrations were present in a particular ratio (N:S of 1.4 or greater) [13]. For context, under the NACE International qualitative categorization of carbon steel corrosion rates, these rates fall in the upper range of moderate general corrosion (0.025–0.12 mm/year) to severe (>0.25 mm/year) [27].

Floating, production, storage, and offloading (FPSO) vessels are often used for offshore oil recovery [21,23]. The topsides of these vessels are equipped with various processing machinery used for separating water, oil, and gas fractions of the produced fluids in preparation for offloading and shipping. The machinery along the flow path of produced fluids, made primarily of carbon steel, is susceptible to both chemical and microbiologically influenced corrosion [21]. The offshore oil reservoirs found in the Jean d’Arc basin, relevant to this study, are high-temperature oil reservoirs reaching in situ temperatures up to 95 °C. The FPSOs involved in oil recovery from these high-temperature reservoirs experience a temperature gradient along the flow path of the injection wells and producer wells, allowing for the growth of microorganisms throughout the system where temperatures conducive to microbial growth exist [23]. The FPSOs that draw from these reservoirs use seawater injection in secondary recovery and operators have used nitrate addition as a method of souring mitigation (personal communication with operators). It is in the interest of offshore oil-producing operators to be able to better predict the success of nitrate/nitrite injection, along with any risk that the use of these chemicals might impart. The use of nitrate or nitrite is less costly and less environmentally damaging in comparison to alternative methods of souring control and MIC mitigation, including the use of biocides [7,8,9,10,11].

In this study, we aimed to elucidate the outcomes of nitrate and nitrite injection in offshore oil recovery from high-temperature reservoirs by observing the microbial and chemical changes, along with the general corrosion rates in a laboratory microcosm experiment using oilfield samples as microbial inoculum. Nitrate and nitrite treatment breakthrough scenarios were established with produced water samples from various sampling locations along the topside flow paths of two FPSO vessels offshore Eastern Canada to assess their risk of corrosion. To date, a survey of topside microbial community compositions combined with their sulfur and nitrogen cycling activity and potential for corrosion capability has not been well documented. Thus, the outcome of this study will add to the breadth of the scientific data available on the effects of nitrate or nitrite injection and inform operators of the offshore oil recovery scenarios on the associated risks with using this method of souring and MIC mitigation.

## 2. Materials and Methods

### 2.1. Samples

**Figure 1:**
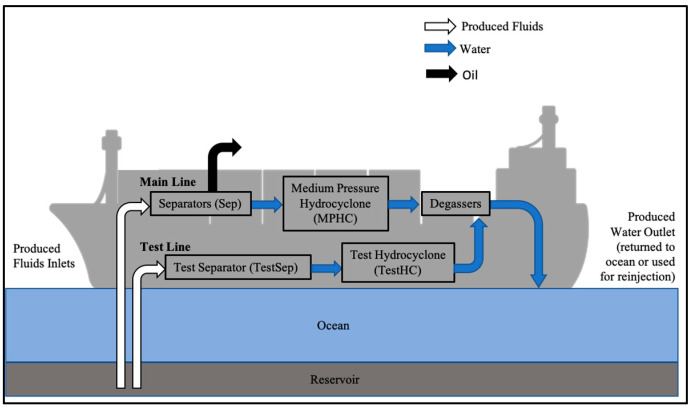
A generalized schematic of the flow path of the produced fluids through an FPSO platform. Both Platforms A and B described in this study follow this general flow path.

The produced water samples that hosted the microbial consortia used in this study were collected from the topsides of two FPSO vessels, designated Platform A and Platform B. The FPSO platforms are located in the Joan d’Arc basin approximately 300 km off the coast of Eastern Canada, and process fluids recovered from the same geographical basin. Produced fluids are processed via a similar path through separation machinery onboard (Figure 1). Platform A represents an oil recovery operation not receiving nitrate treatment for souring, while portions of the reservoir fluids processed by Platform B have received nitrate treatment. The produced water from each sampling location described in this study was collected into sterile 1 L Nalgene bottles, sealed without headspace, and shipped under cold conditions to the University of Calgary within 10 days of sampling. Upon receipt of the produced water samples, 10 mL were collected and stored at −20 °C for chemical analyses, and 200 mL were used for collecting biomass by filtration using a 0.4 μm vacuum filtration system (Nalgene^TM^ RapidFlow^TM^, ThermoFisher, Waltham, MA, USA). The filter was aseptically removed and stored at 4 °C with 1 mL of DNAzol^®^ (ThermoFisher, Waltham, MA, USA) for nucleic acid preservation. The remainder of each sample was used for microcosm assembly.

### 2.2. Microcosm Assembly

Anoxic microcosms were constructed using 120 mL serum bottles containing 50 mL of produced water sample from 3 sampling locations from Platform A and Platform B (Figure 1). The microcosms contained a headspace of 90% N_2_ and 10% CO_2_ and 1 corrosion coupon (1 cm × 1 cm) composed of the same material used in the topside machinery (A333 Grade 6 carbon steel, Metal Samples, Munford, AL; preparation described below). A total of 4 treatments were applied to each location sample: (1) no additive, designated ‘NA’; (2) 5 mM nitrate, designated ‘Nitrate’; (3) 5 mM nitrate and 2 mM sulfide, designated ‘S_nitrate’; or (4) 5 mM nitrite and 2 mM sulfide, designated ‘S_nitrite’. These treatments were chosen to be representative of: (1) an untreated condition, (2) a nitrate injection condition, (3) a nitrate injection condition in a sour system, and (4) and a nitrite injection condition in a sour system, respectively. When nitrate was added to injection water, breakthrough concentrations (i.e., the concentration in the produced fluids after having passed through the reservoir) have been reported to range between 0.5 mM and 5 mM [26,28]; 5 mM was chosen as the concentration of nitrate or nitrite to test in these experiments. Anoxic sterile stock solutions of Na_2_S, NaNO_3_, and NaNO_2_ were prepared and added to sealed anoxic microcosms via a sterile N_2_-flushed needle and syringe to reach the appropriate concentrations. Microcosms were not amended with any additional nutrients or external carbon sources, instead relying on the carbon dioxide present in the headspace (10% CO_2_), the acetate content in the sample (Table 1), and any residual hydrocarbons or other organic compounds that may have been present in the produced water (not determined). Each experimental condition was established in triplicate, along with one sterile control microcosm (sterilized by autoclaving); a limited volume of each sample only permitted the establishment of a single sterile control incubation for each condition. Microcosms were incubated at 54 °C, the temperature of most of the sampling points of the topside machinery, for 156 days with light shaking (80 rpm). Sampling over time for chemical analyses was performed using sterile N_2_-flushed needles and syringes.

### 2.3. Corrosion Coupon Preparation and Analysis

Carbon steel corrosion coupons (A333 Grade 6 carbon steel) were cut into 1 cm × 1 cm pieces, and were then treated with an industry standard protocol [27]. Briefly, the coupons were prepared prior to the microcosm assembly by polishing using silicon carbide papers, increasing sequentially from 400 to 800 grit paper. The coupons were then cleaned with deionized water followed by acetone, then dried under a stream of N_2_. The coupons were weighed three times using an analytical scale and the average was recorded as the time-zero coupon weight. The coupons were then fixed to a nylon string using an epoxy resin, which coated the entire surface area of one 1 cm × 1 cm face of the coupon, leaving the other face of the coupon exposed. The other end of the nylon string was threaded into a butyl rubber stopper that was then used to seal the anoxic microcosm. After 156 days of incubation, the coupons were removed from the serum bottles. For each condition (tested in triplicate), one coupon from one replicate was used for microscopic imaging prior to cleaning (see below), while the other two coupons from the remaining two replicates were used for microbial community analysis (see Section 2.5) by swabbing for sessile biomass using a sterile cotton swab. Following imaging or swabbing, the coupons were cleaned according to a standard practice protocol to remove corrosion products [27]. The coupons were then weighed three times on an analytical scale and the average was used as the final coupon weight. The weight-loss value was used to calculate the general corrosion rate by the following equation: *CR* = 87,600 × Δ*W/A* × *T* × *D*, where *A* is the exposed surface area of the coupon (1.4 cm^2^), *T* is the incubation time (3696 h), and *D* is the density of the carbon steel (7.86 g/cm^3^) [27]. 

The clean coupons were then imaged using an Olympus SZ61 light microscope (Tokyo, Japan). Light microscopy images of the clean corrosion coupons were analyzed using a grid system (grid of 64) to assign pitting density as a result of the different treatments. This approach was used as a low-cost metric to determine the frequency of pitting in live and sterile incubations. One replicate corrosion coupon from the NA and S_nitrate conditions for each sampling location was removed from the serum bottle and preserved for scanning electron microscopy (SEM) imaging using a previously described protocol [29]. The biofilms on the surface of the corrosion coupons were imaged using a field emission SEM (FEI Quanta 250 FEG, Thermo Fisher Scientific, Waltham, MA, USA). 

### 2.4. Water Chemistry

Sulfate, nitrate, and nitrite concentrations were measured by high-performance liquid chromatography (HPLC) using an IC-PAK^TM^ anion column HC (150 × 4.6 mm, Waters Corp, Milford, MA, USA) and eluted with 2% sodium borate-gluconate, 12% acetonitrile, and 2% butanol buffer at a flow rate of 2.0 mL/min. Sulfate was detected using a Waters 432 conductivity detector, and nitrate and nitrite were detected using a Waters 2489 UV/Visible detector set at 200 nm. Samples were prepared for ion chromatography by centrifuging at 14,000 rpm for 5 min to remove debris, then 100 μL of the sample supernatant were added to 400 μL of the elution buffer described above. Calibration standards were prepared using known concentrations (2–10 mM) of nitrate (NaNO_3_), nitrite (NaNO_2_), and sulfate (Na_2_SO_4_) that were analyzed using the same method. The volatile fatty acids (VFAs) acetate, propionate, and butyrate were measured using a Waters 600E HPLC (Waters Corp, Milford, MA, USA) equipped with a Waters 2487 UV detector set to 210 nm, and a Prevail organic acid 5u column (250 × 4.6 mm, Alltech, Guelph, ON, Canada). A flowrate of 1.0 mL/min was used, with 25 mM KH_2_PO_4_ (pH 2.5) as the eluent. Samples for VFA analysis were prepared by centrifugation at 14,000 rpm for 5 min, followed by acidification with 20 μL of phosphoric acid (1 M) to 300 μL of sample supernatant. Sulfide, ammonium, and Fe^2+/3+^ concentrations were measured spectrophotometrically using previously described protocols [30,31,32]. Produced water salinities were determined as the molar equivalent of NaCl, by measuring conductivity using an Orion™ Versa Star™ advanced electrochemistry meter (Model 013005MD, Thermo Fisher, Waltham, MA, USA). The pH was measured using the pH channel on the same instrument. The ATP measurements were made on initial produced water samples as an estimation of the number of active microbial cells using the Quench-Gone Organic Modified™ kit from LuminUltra Technologies Ltd. (Fredericton, NB, Canada). GraphPad Prism software (GraphPad, San Diego, CA, USA) was used to perform unpaired t-tests to assign significance in chemistry data sets. Data with *p*-values higher than 0.05 were considered insignificant.

### 2.5. Microbial Community Analysis

Microbial community analysis was performed upon receipt of the produced water samples (time zero), and at the experiment termination (end point). The end-point microbial community analysis was performed on the sessile (coupon-attached) biomass. The sessile biomass was collected by swabbing the surface of corrosion coupons (1 cm by 1 cm surface area) immediately upon removal from a microcosm using a sterile cotton swab moistened with sterile phosphate buffer [33]. DNA was extracted from biomass samples using the FastDNA Spin Kit for Soil (MP Biomedicals, Solon, OH, USA) following the manufacturer’s instructions. DNA sequencing was performed using Illumina MiSeq sequencing technology. The amplification of the V4-V5 variable region of the 16S rRNA gene was performed using the forward primer Illumina515f (GTGYCAGCMGCCGCGGTAA) and the reverse primer Illumina926r (CCGYCAATTYMTTTRAGTTT), in a two-step polymerase chain reaction (PCR). First-round PCR amplification reactions were carried out using either Fermentas Taq polymerase (ThermoFisher, Waltham, MA, USA) or KAPA Hi-Fi polymerase (Roche, Basel, Switzerland), and second-round indexing amplification was carried out using Fermentas Taq polymerase. The PCR reactions using Fermentas Taq polymerase consisted of 25 μL of polymerase mastermix; 1 μL of each forward and reverse primer (10 pM); 5, 8, or 10 μL of template DNA; and PCR grade water to make up the final volume of 50 μL. The PCR reactions using KAPA polymerase consisted of 12.5 μL of polymerase mastermix; 0.5 μL of forward and reverse primers; 2, 5, or 8 μL of template DNA; and the remaining volume for a 25 μL reaction made up with PCR grade water. The PCR was performed on a ProFlex PCR System (Invitrogen) thermocycler using the following program: 95 °C for 5 min, 95 °C for 30 s, 55 °C for 4 min, 72 °C for 2 min, and 72 °C for 10 min. Steps 2–4 were repeated 25 times. The purification of the first- and second-round PCR products was performed using either AMPure Magnetic Beads (Beckman Coulter, Brea, CA, USA), or with a Zymo Select-a-Size DNA Clean and Concentrator Kit (Irvine, CA, USA). The samples were normalized and IlluminaMiSeq v3 600 cycle sequencing was outsourced to the Centre for Health Genomics and Informatics (University of Calgary, Cumming School of Medicine). The sequencing results were processed using QIIME 2019.7 software. DADA2 was used for denoising the data (forward reads were truncated at 280 nucleotides, reverse reads at 260 nucleotides, with a 20-nucleotide overlap) and taxonomy was assigned using the SILVA 132 database. The outputs from QIIME2 processing are classified as amplicon sequence variants (ASVs). β-diversity analysis was performed on data using ‘vegan’ and ‘picante’ scripts in R. 

## 3. Results

### 3.1. Produced Water-Sample Analysis

#### 3.1.1. Chemical Analysis of the Produced Water Samples

Produced water samples were collected from three locations on the topsides of two FPSO vessels. Figure 1 shows a schematic of the general flow of the produced fluids through the FPSO machinery. On both FPSOs (Platform A and Platform B), there is a main line, where produced fluids from many different wells are combined into a common pipeline and flow throughout the main-line machinery. There is also a test line, where operators can draw produced fluids from one specific well, in order to perform testing on a well of interest. The samples used in this experiment were retrieved from the medium-pressure separator (A_MPSep), medium-pressure hydrocyclone (A_MPHC), and test hydrocyclone (A_TestHC) from Platform A, and the medium-pressure hydrocyclone (B_MPHC), test separator (B_TestSep), and test hydrocyclone (B_TestHC) from Platform B. This onboard machinery is used to separate oil, gas, and water fractions, and to remove debris from the water fraction prior to disposal or reuse. The salinity of the collected samples ranged between 0.47–0.57 M NaCl, typical for that of seawater (Table 1). The pH of the water samples from both platforms, measured upon receipt, was approximately neutral (7.0–7.9, Table 1). Sulfate concentrations in Platform A samples were between 8.1 mM and 9.3 mM, noticeably lower than the Platform B samples, which had sulfate concentrations ranging between 19.4 mM and 23.8 mM. The discrepancy in sulfate concentrations between the platform samples may be explained by a mixing effect, wherein the mixing of injected seawater with the formation waters of lower sulfate concentrations yielded an overall lower sulfate concentration compared to that of seawater. Platform A experienced more mixing with formation waters compared to Platform B, likely explaining the reason for the lower sulfate concentration in Platform A fluids (personal communication with operators). Iron was not detected in the Platform A samples, and was minimal in Platform B samples, with each location sample falling equal to or below 0.6 mM Fe^2+/3+^, while ammonium concentrations were similar across the two platforms (1.2–1.6 mM). Acetate concentrations were approximately three times higher in Platform A samples compared to Platform B samples (Table 1), and neither propionate nor butyrate were detected in any of the produced water samples (not shown).

#### 3.1.2. Microbial Analysis of the Produced Water Samples

**Figure 2:**
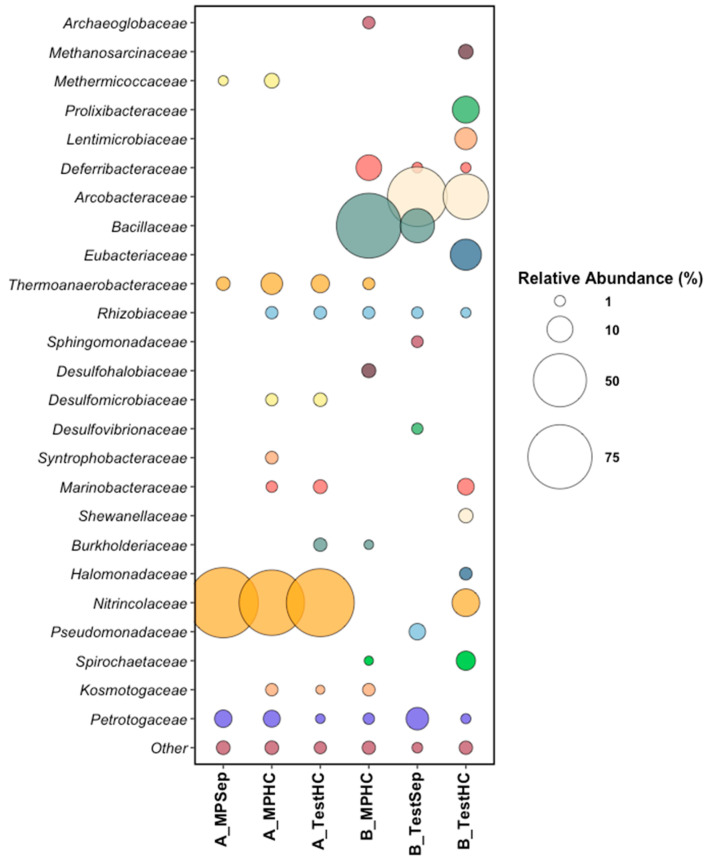
16S rRNA gene sequencing data displayed by relative sequence abundance (%) for sampling locations on Platforms A and B. Only taxa above a 1% relative sequence abundance are displayed. The ‘other’ category includes taxa that are below a 1% relative read abundance. If no bubble appears next to a taxon, it indicates that that taxon does not appear above a 1% relative sequence abundance in the corresponding sample.

Samples from Platform A contained 10^4^–10^5^ active cells per mL, while Platform B contained 10^6^ active cells per mL in all location samples (Table 1). Microbial community analysis of the produced water samples from the six sampling locations at the time of incubation assembly (time 0) revealed the presence of diverse microbial taxa (Figure 2). *Nitrincolaceae* was the dominant taxon in Platform A samples at an average of 85% relative read abundance across the three sampling locations. *Nitrincolaceae*, recently detected in marine phytoplankton blooms [34], are members of *Oceanospirillales*, an order containing members capable of aerobic hydrocarbon degradation in seawater [35]. Platform A samples harbored sulfate-reducing *Desulfomicrobiaceae* at 1.5% and 1.9% relative abundances in the main-line and test-line hydrocyclones, respectively, indicating the potential for biological sulfate reduction in the associated microcosms. *Kosmotogaceae* and *Petrotogaceae*, taxa that contain fermentative members, made up an average of 1.6% relative read abundance in Platform A. *Kosmotogaceae* and *Petrotogaceae* have been found previously associated with oilfield brine and high-temperature petroleum reservoirs [36,37]. *Thermoanaerobacteraceae* was present in all three samples collected from Platform A ranging from 2.0% to 6.5% relative read abundances, and is a common high-temperature oilfield taxon with members capable of the fermentation of or reduction in thiosulfate to produce sulfide [38,39].

Microbial community analysis of samples from Platform B revealed *Bacillaceae* and *Arcobacteraceae* as the dominant taxa. Though most taxa are common to all three of the Platform B sampling locations, the main line had *Bacillaceae* as the dominant taxon at 77% relative read abundance, while both of the test-line samples (B_TestSep and B_TestHC) revealed *Arcobacteraceae* to be dominant at between 36% and 65% relative read abundances. *Arcobacteraceae* are widespread in many aquatic environments, including in oilfield-produced water, with members having the ability to reduce nitrate, oxidize sulfide, and produce elemental sulfur [40,41,42]. Notably, Platform B samples harbored *Deferribacteraceae*, present between 1% and 10% relative read abundances; members of this taxon have been detected in thermogenic oilfield fluids [43] and in offshore-produced water in nitrate-treated systems [26,44].

### 3.2. Effects of Nitrate or Nitrite Treatment on Sulfate Reduction

**Figure 3:**
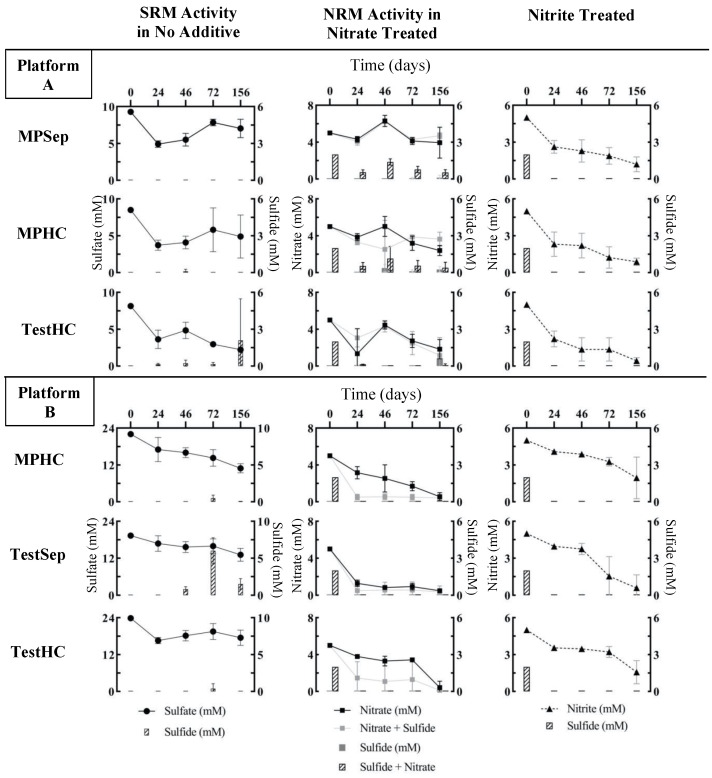
Sulfate, sulfide, nitrate, and nitrite concentrations (mM) in the microcosms over time for Platform (**A**) (top) and (**B**) (bottom) microcosms. The left panel shows the activity of SRMs as determined by sulfate loss and sulfide production over time. The center panel shows the activity of NRMs as determined by nitrate loss over time, and either sulfide production (in nitrate only treatments) or sulfide loss (in S_nitrate treatments where SOM may be active). The data shown in gray corresponds to the nitrate-only treatment, while the data shown in black corresponds to the S_nitrate treatment. The right panel shows the nitrite loss and sulfide loss/production over time. Sulfate (circles), nitrate (squares), and nitrite (triangles) trends are displayed as a continuous line graph, and the sulfide data are displayed as bars. The lack of a bar indicates little or no sulfide. The data points indicate the means of the triplicate incubations, and the error bars represent standard deviation of the mean.

**Figure 4:**
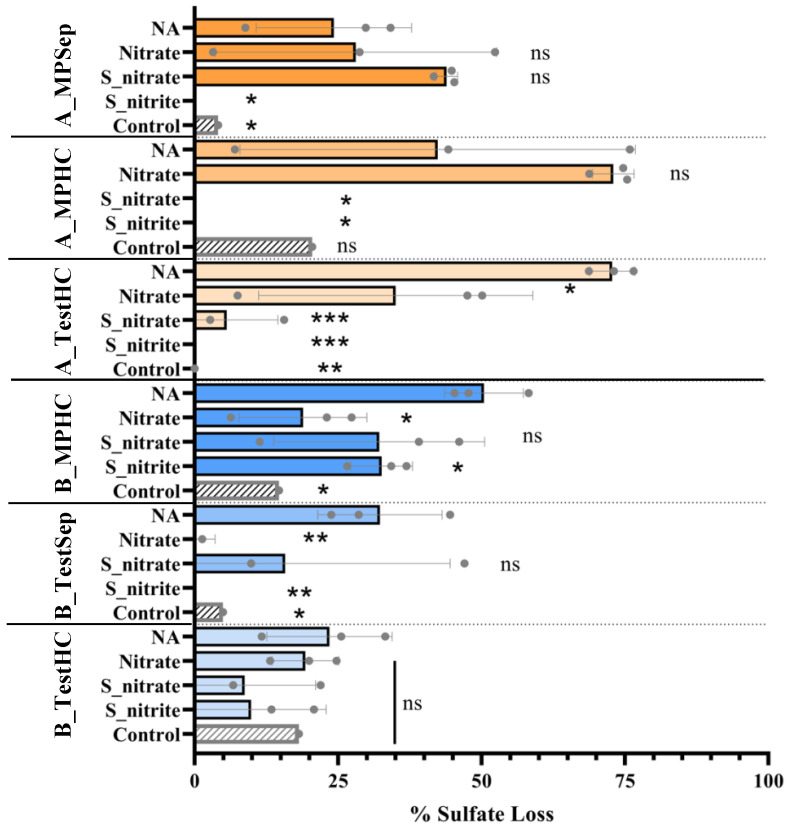
Percent sulfate loss over a 156-day incubation period under different treatment scenarios. Platform A samples are in orange, and Platform B samples are in blue. The data points (gray circles) indicate the values of each replicate, and the error bars indicate the mean among 3 replicates (*n* = 3). The asterisks indicate the *p*-value (*, 0.033; **, 0.002; ***, <0.001; ns, not significant) of a treatment compared to its corresponding no-additive (NA) microcosms. ‘Control’ = sterile control.

Sulfate, sulfide, nitrate, and nitrite concentrations were monitored throughout the 156-day experiment to observe the impact of nitrate or nitrate treatments on souring (sulfate reduction). Figure 3 shows SRM activity in no-additive microcosms (NA) by the analysis of sulfate and sulfide, NRM activity in nitrate-treated microcosms (Nitrate, S_nitrate) by the analysis of nitrate and sulfide, and the loss of nitrite in nitrite-treated microcosms. The A_TestHC microcosms showed the most sulfate reduction, with approximately 73% of sulfate loss over the course of the 156-day incubation compared to the Platform A main-line location samples which had 24% (A_MPSep) and 42% (A_MPHC) sulfate losses (Figure 4). The A_TestHC sulfate loss was coupled with sulfide accumulation up to 2.1 ± 3.4 mM at 156 days. Microcosms prepared from all Platform B sampling locations also showed sulfate loss over the course of incubation, with the B_MPHC microcosms having had roughly 20% more sulfate loss compared to the test-line microcosms (B_TestSep, B_TestHC). Platform B microcosms showed the maximum sulfide accumulation at the 72-day sampling point, with the highest sulfide accumulation in B_TestSep microcosms, reaching 6.0 ± 1.8 mM (Figure 3, left panel).

In the nitrate-treated microcosms (Figure 3, middle panel), Platform B samples showed a greater extent of nitrate reduction over time in both the Nitrate and S_nitrate treatments, where all sampling location microcosms had over 90% nitrate loss. In contrast, a maximum of 68% nitrate loss was observed in the Platform A location samples (A_TestHC). The nitrate reduction in Platform B microcosms was coupled with the complete oxidation of sulfide by day 24 in the S_nitrate treatments. A similar trend was observed in only the A_TestHC location of Platform A. The Platform A mainline sample microcosms treated with S_nitrate showed a more gradual sulfide loss over time with minimal nitrate reduction observed (approximately 6% nitrate loss). Additionally, microcosms from Platform B treated with nitrate only had no sulfide production by the end of the incubation, while Platform A microcosms showed sulfide accumulation as early as 46 days into the incubation period. Nitrite accumulation was observed in Platform B microcosms when nitrate was added (Table 2), while nitrite was not detected in Platform A microcosms (not shown).

In S_nitrite-treated microcosms from all samples, nitrite concentrations gradually decreased over the course of incubation, and complete sulfide loss was observed by the first sampling point (24 days) (Figure 3, right panel). In most sampling location microcosms from Platforms A and B, there was little or no sulfate loss, with the exception of the MPHC of Platform B (B_MPHC) microcosms (Figure 3). In the first 24 days, there was a lack of efficacy in sulfate reduction inhibition as sulfide was detected at close to the same level added (~2 mM, Figure 3, right panel). This initial observation was likely due to the chemical reactivity of nitrite and sulfide, where nitrite is converted to ammonium abiotically [45] (Equation (1)). Once the stoichiometric amount of nitrite was chemically reduced to ammonium (~0.5 mM nitrite, based on 4 moles HS^−^: 1 mole NO_2_^−^, Equation (1)), approximately 4.5 mM nitrite should remain. At day 24, approximately 4 mM nitrite was experimentally measured, and subsequently decreased over the 156-day incubation period. After 24 days, no additional sulfide was detected, suggesting the efficacy of nitrite in inhibiting sulfate reduction.

(1) $$4HS^{-}+NO_{2}^{-}+5H^{+}\;↔\;\;HS^{-}+3S^{0}+NH_{4}^{+}+2H_{2}O$$

A summary of the sulfate loss in all microcosms due to biological sulfate reduction after the 156-day incubation period is shown in Figure 4. The microcosms with no souring treatment applied (NA) revealed the most sulfate loss in A_TestHC, B_MPHC, and B_TestSep, ranging between 32.3 ± 10.8% and 72.8 ± 3.9% sulfate losses. The 5 mM nitrate-treated microcosms (Nitrate) had less sulfate loss than the no additive in A_TestHC, B_MPHC, and B_TestSep. When sulfide was added along with the 5 mM nitrate treatment (S_nitrate), there was no statistically significant sulfate loss compared to the no additive (NA) in samples from Platform B, however the A_MPHC and A_TestHC microcosms did show significant sulfate reductions. Finally, the sulfide- and nitrite-treated microcosms (S_nitrite) showed significant sulfate reduction inhibition compared to the no additive in all sampling locations, except for B_TestHC (Figure 4). While variability was observed, the overall results showed that the presence of nitrite was more effective than nitrate treatment in inhibiting sulfate reduction in the microcosm tests.

### 3.3. End-Point Microbial Community Analysis

**Figure 5:**
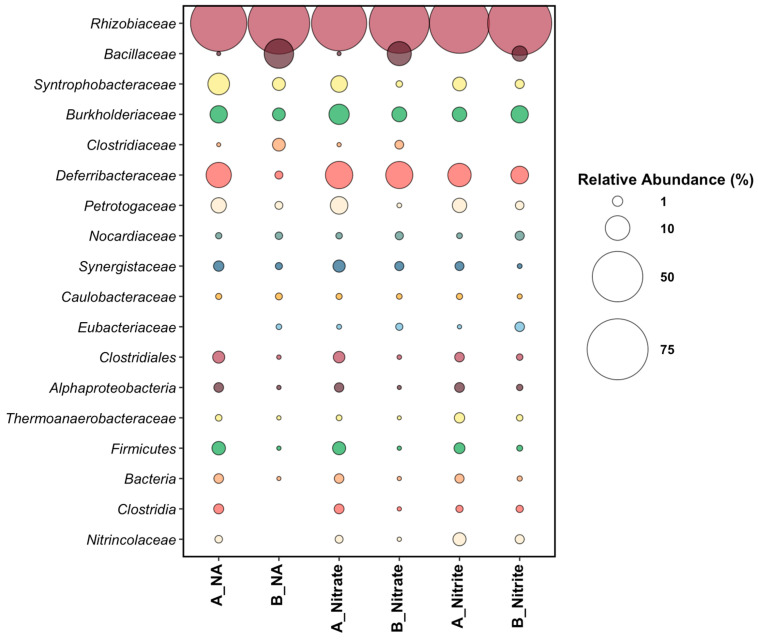
Relative sequence abundance of taxa above a 1% average relative read abundance across all sampling locations from the 16S rRNA gene sequencing data of Platform A and B microcosms across treatments. Taxa are listed by family, or by the lowest taxonomic output from the Silva-132 database.

Microbial community composition was analyzed in all treatments at the end of the 156-day incubation period. An end-point-only analysis was performed to ensure that enough time had passed to observe potential microbial corrosion under the different treatments, and to minimize substantial disruption to the incubations. Further, a microbial community analysis of the sessile (coupon-attached) population was only possible at the end of the incubation period. Figure 5 displays the microbial community analysis data from 16S rRNA gene sequencing of the sessile organisms that were established on the surface of the corrosion coupons in the experimental microcosms.

*Rhizobiaceae* dominated in microcosms from both platforms and across all treatments. The saprophytic nature of the microbial members of this family could be a reason for its high relative abundance in the end-point sequencing of this high-temperature, long-term experiment. Some members of the *Rhizobiaceae* are capable of anaerobic growth wherein nitrate is reduced to nitrite [46]. Additionally, some strains have been found to be halotolerant [47]. At the end of the microcosm experiment, *Deferribacteraceae* was present across both platforms and all treatments. Compared to its time-zero microbial community data (Figure 2), Platform A experienced the greatest increase in relative read abundance for *Deferribacteraceae* (approximately 8% across all Platform A treatments). For Platform B samples, *Deferribacteraceae* maximally increased in relative read abundance in the nitrate treatments (12.5% relative read abundance), followed by the nitrite treatment (4.5% relative read abundance), and lastly in the no-additive condition (0.4% relative read abundance) (Figure 5). There was disparity between the relative abundances of *Bacillaceae* in Platforms A and B, where Platform B had an average relative read abundance range of 3.1% (nitrite treated) to 9.5% (nitrate treated), while Platform A had less than 0.003% relative read abundance. *Syntrophobacteraceae* was present across both platforms and all treatments, wherein its maximum relative read abundance was observed in the NA treatment (7.4% and 2.0% relative read abundances for Platforms A and B, respectively). *Clostridiaceae* was detected in samples from both Platforms in each treatment, except in the nitrite-treated samples (Figure 5).

### 3.4. General Corrosion Rates and Pitting Counts

**Figure 6:**
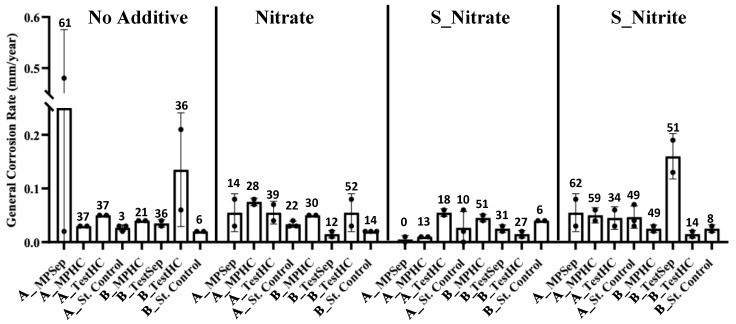
General corrosion rates calculated for the coupons in microcosms from each sampling location and treatment. The bars represent the mean general corrosion rates of two replicates (*n* = 2) and the error bars represent the standard error between replicates. The number displayed above each bar is the highest pitting frequency observed among the replicates in the corresponding treatment and sampling locations.

Figure 6 displays the average general corrosion rate of each treatment investigated in this study. Overall, a high variability in the corrosion rates across the replicates was observed. The no-additive (NA) microcosms experienced the highest average corrosion rates, followed by the S_nitrite-treated microcosms. In particular, a replicate from the NA A_MPSep had the highest general corrosion rate measured in this study, at 0.48 mm/year. This corrosion rate is classified as severe, according to the NACE qualitative general corrosion rate scale [27]. The B_TestSep microcosms treated with sulfide and nitrite (S_nitrite) had an average corrosion rate of 0.16 mm/year, and the Platform A S_nitrite-treated samples had an average corrosion rate of 0.05 mm/year, both falling within the moderate range of corrosion [27]. The nitrate-only treatment displayed moderate corrosion rates between 0.07–0.08 mm/year in at least one replicate for each sampling location. The nitrate and sulfide treatments (S_nitrate) revealed low corrosion rates for Platform A and B microcosms with the exception of A_TestHC and B_MPHC (0.06 mm/year and 0.05 mm/year, respectively). The corrosion rates of sterile controls for Platform A microcosms with no additive (NA) and the nitrate treatment (N) remained below the live replicates. The A_MPSep and A_MPHC live replicates treated with S_nitrate had lower corrosion rates than their corresponding sterile control. In contrast, the S_nitrite sterile control for Platform A samples had corrosion rates comparable to the live replicates. One replicate from the sterile controls of Platform B samples (B_TestSep) indicated corrosion rates above 0.15 mm/year. Given that the corresponding live replicates did not also have accelerated corrosion rates, it is hypothesized that autoclaving (high temperature and pressure) may have altered an unknown chemical additive in the produced water to produce a corrosive by-product.

The NA microcosms from Platforms A and B had variable pitting counts, and at least one replicate from each location had a >35 pitting count. The nitrate-only treatments resulted in moderate pitting counts relative to the NA microcosms, while the S_nitrate microcosms exhibited low pitting counts. Finally, the S_nitrite-treated microcosms had high pitting counts in both experimental replicates and sterile controls in all but two locations from Platform B (B_MPHC and B_TestHC).

**Figure 7:**
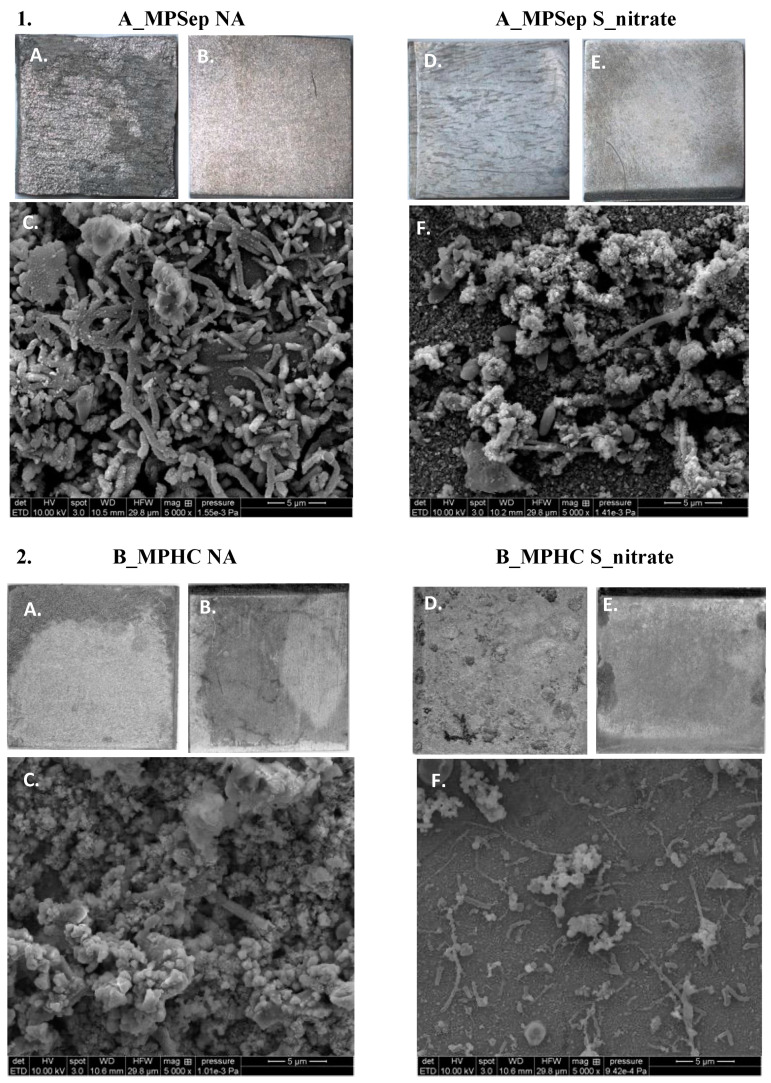
Scanning electron microscopy (SEM) and light microscopy images of the surface of the select corrosion coupons from one sampling location on each platform (Platform A—**1**, Platform B—**2**) comparing the no-additive treatment (NA) and the nitrate plus sulfide treatment (S_nitrate). (**A**,**B**,**D**,**E**) display the light microscopy images (coupon dimensions are 1 cm × 1 cm), while (**C**,**F**) display the SEM images (5000× magnification) in subfigures **1** and **2**.

Light microscopy and SEM were performed on the carbon steel corrosion coupons to observe corrosion, the presence of microbial cells, and any corrosion products or extracellular polymeric substances on the coupon surfaces (Figure 7). The microscopic imaging for A_MPSep NA and S_nitrate treatments, and the B_MPHC NA and S_nitrate treatments are shown as examples. The surface of the A_MPSep NA coupon (1A) had visible degradation on its surface, compared to its sterile control coupon (1B). The SEM image of the same location sample and treatment (1C) showed the presence of microbial cells as a biofilm on the coupon surface. The S_nitrate-treated coupons of the same sample location had less degradation on the surface of the coupon (1D), compared to its sterile control (1E). The SEM image for this treatment also revealed fewer microbial cells on the surface of the coupon (1F). Similar results were observed in the Platform B coupons for the no-additive treatment (NA), however the nitrate plus sulfide treatment (S_nitrate) coupons showed signs of degradation in both the experimental replicate and the sterile control (2D, 2E). The SEM image of the coupon from this treatment did, however, indicate a sparse coupon surface with few microbial cells and little corrosion product (2F).

## 4. Discussion

The use of nitrate or nitrite to inhibit oilfield souring during secondary recovery operations has been successful in some crude oil operations [26,28]. However, several laboratory studies, primarily conducted under mesophilic conditions with microbial enrichments or pure cultures, have shown that some byproducts of this treatment approach are corrosive to metal infrastructures [9,10,13]. Thus, determining the potential for effective souring control using nitrate or nitrite while also assessing potential infrastructure corrosion on the topsides of offshore platforms is requiredto ensure safe and economic energy recovery operations. In particular, assessment studies that use oilfield samples and mimic infrastructure-operating temperatures are needed as few exist [21]. 

Here, the application of nitrate and nitrite treatments to inhibit sulfate reduction and their effect on metal corrosion under thermophilic conditions (55 °C) was assessed using produced water samples collected from the topsides of two different FPSO platforms. We found that microbial communities inhabiting the topside machinery of these two different FPSO platforms reacted differently to nitrate and nitrite treatments for souring. Given the previous research showing nitrite accumulation at temperatures >50 °C when nitrate is used to treat souring [23,24], we expected nitrite accumulation in the 55 °C microcosms. Overall, the microbial community members in Platform B samples reacted as expected to the nitrate treatment, wherein nitrate (added at 5 mM) effectively inhibited sulfate reduction and, consequently, sulfide production (Figure 3 and Figure 4). In Platform B nitrate-treated microcosms, nitrite accumulated variably between the sampling locations (ranging between 0.29–2.45 mM), which presumably aided in sulfate reduction inhibition. In contrast, the microbial community members in the Platform A samples did not react as expected to the nitrate treatment. Platform A samples treated with nitrate showed comparatively less inhibition of sulfate reduction, as sulfide production was not curtailed (Figure 3 and Figure 4). Nitrite did not accumulate in Platform A nitrate-treated microcosms. The associated corrosion rates from these samples were all within an error of one another between the two platforms, indicating no significant difference in the corrosion risk due to nitrate treatment, and sterile control corrosion rates were also similarly low (Figure 6). 

In contrast, nitrite treatment (nitrite added at 5 mM) was successful in inhibiting sulfate reduction by microbial community members inhabiting various locations on the topsides of both Platforms A and B. This result is consistent with the previous findings asserting that the efficacy of nitrate treatment depends on the growth and activity of the extant nitrate-reducing microbial population, whereas nitrite treatment works to directly inhibit the dissimilatory sulfite reductase in SRMs [22,48]. It has also been found that adding nitrite to injection water is more effective than other treatment chemicals in oilfields, including in high-temperature scenarios [49]. Modeling simulations based on several case studies asserted that nitrite is the main inhibitory chemical in nitrate-treated sour oilfield systems [50]. In the present study, the produced fluid samples were from a high-temperature reservoir that can reach temperatures of up to 90 °C. The mode of action of nitrite in sulfate reduction inhibition, in addition to its efficacy in high-temperature oil fields, may be the reason that nitrite performed better across both platform samples for reducing sulfate reduction, as opposed to the variability in efficacy of the nitrate treatments [23,24,50]. The corrosion rates (assessed after the 156-day incubation) measured for the nitrite-treated microcosms in our study indicate a low corrosion risk (Figure 6), however the potential chemical corrosivity of nitrite can be observed in the nitrite-treated sterile controls that showed a moderate corrosion rate of 0.07 mm/year [27].

**Figure 8:**
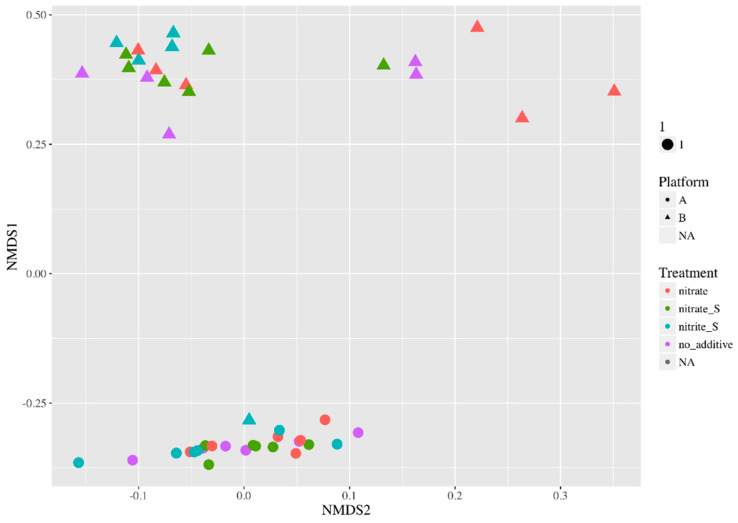
Non-metric multidimensional scaling (NMDS) plot describing the Bray–Curtis dissimilarity between 16S rRNA gene sequencing DNA libraries of Platforms A (circles) and B (triangles), and various treatments (differentiated by color). Each data point represents the microbial community present in one microcosm after the 156-day incubation.

The variability in the chemical results of the nitrate-treated microcosms is likely due to differences in the microbial community compositions in the Platform A and B topside samples and the prior treatment history of the oilfield samples being processed on these FPSO. A non-metric multidimensional scaling plot of the 16S rRNA gene sequencing data following the incubations under various treatment scenarios reveals the distinction between the microbial communities associated with the Platform A and B topside infrastructure. This analysis shows that Platform A samples cluster together and distantly from Platform B samples (Figure 8), indicating a difference in the microbial community structure.

Platform B microcosms showed nitrate reduction paired with sulfide oxidation over time in all sampling locations treated with nitrate and sulfide (Figure 3), presumably indicating the successful stimulation of NR-SOB. Notably, two of the three Platform B samples used to prepare the incubations contained high relative abundances of *Arcobacteraceae* (Figure 2), some members of which are known NR-SOB [51,52], or are able to reduce nitrate to ammonium via the dissimilatory nitrate reduction pathway under moderate salinity conditions [41]. Platform B samples also initially harbored members of the *Deferribacteraceae* (between 1–10% relative abundance), a taxon commonly found in nitrate-treated offshore wells and a presumed nitrate reducer [26,28,44]. The comparatively high relative abundances of nitrate reducers (heterotrophic or sulfide-oxidizing) found in the topside samples collected from Platform B aligned with the treatment history of the oilfield, wherein some parts of the oilfield processed by this platform had a history of nitrate treatment for souring. The exposure of the microbial communities associated with the oil recovery operation to nitrate presumably favored the growth and activity of nitrate-reducing taxa, a phenomenon also observed with water chemistry changes across an entire oilfield in the Danish North Sea [26]. Sulfate reduction was inhibited in both nitrate-treated scenarios (in the presence or absence of sulfide) in the Platform B incubations. 

In contrast, microbial communities in the Platform A samples generally responded to nitrate treatment with a slower rate of nitrate reduction, gradual sulfide loss over the course of incubation in microcosms with added sulfide, and sulfide production in microcosms receiving the nitrate-only treatment. In contrast to Platform B, the samples collected from Platform A topsides did not reveal the presence of NR-SOB, such as *Arcobacteraceae*, in the time 0 analysis (Figure 2) or other nitrate-reducers, such as *Deferribacteraceae*, which may help to explain the lower efficacy of nitrate treatment in the Platform A microcosms. The A_TestHC location chemical trends appeared more similar to that of Platform B samples, where sulfide utilization was coupled with nitrate reduction; however, in the nitrate-only treatment, sulfide production was still observed (Figure 4). This result indicates that the nitrate treatment tested here did not work as effectively for Platform A as it did for Platform B, likely due to the lack of appropriate microbial community members able to couple nitrate reduction with sulfide oxidation. The trends in microbial activity observed in the Platform A microcosms align with the operational history of the oilfield served by Platform A, wherein nitrate has not been applied to the field.

In the microbial community analysis of coupon-attached consortia after incubation, the normalization of microbial community structure was observed between treatments in samples from the same platform (Figure 5). This effect may have been due to the long-term incubation (156 days) where the only treatment dose was at time zero. This incubation time was chosen as to avoid over estimating corrosion rates due to short incubation, to minimize microcosm disturbance, and to allow full chemical trends to be observed. However, without replenishing carbon sources, electron acceptors, vitamins, and trace elements, we hypothesize that only resilient taxa survived the high-temperature conditions for the full 156-day incubation, while others, which may have played a role in the observed chemical trends earlier in the incubation period, did not. Despite this finding, some details of the microbial community analysis are important for interpretation of the chemical data. *Deferribacteraceae* was observed across all treatments and in samples from both Platforms in varying relative read abundances (Figure 5). *Deferribacteraceae* has microbial members capable of using iron or nitrate as electron acceptors [28,53], which may explain its presence across all microcosms, given the availability of nitrate (nitrate, and S_nitrate treatments) or iron from the corrosion coupons (in non-nitrate treatments). Though some iron-reducing bacteria have previously been shown to play a role in MIC [16,17] or detected on infrastructure experiencing corrosion [19], any specific involvement of *Deferribacteraceae* in corrosive biofilms is not presently known. Members of the *Clostridia* were present in no-additive and nitrate-treated microcosms, possibly playing a role in biofilm formation on the surface of corrosion coupons [54]. Nitrite appeared to prevent the growth of *Clostridia* as part of the sessile community, as members of this taxon were not detected in the nitrite-amended incubations at the end of the incubation period (Figure 5); the reasons for this observation are not clear. *Syntrophobacteraceae*, some members of which are capable of sulfate reduction and have been found in corrosive biofilms [43,55], was present in all microcosms across all treatments, however Platform A coupons contained a noticeably higher relative abundance of this taxon compared to the coupons retrieved from the Platform B microcosms. The higher relative abundance of this taxon in Platform A is consistent with the sulfide concentrations observed in nitrate-treated microcosms, where sulfide was produced in Platform A microcosms but not in Platform B microcosms. *Bacillus* was enriched across all treatments in Platform B samples. Some *Bacillus* species have been reported to be involved in MIC through mechanisms of biofilm formation [56], however there are also certain strains that have an inhibitory effect on MIC by way of biosurfactant production [57,58,59]. This presence of *Bacillus* may have worked to inhibit microbial corrosion in Platform B samples, but further research would be needed to confirm this and the specific metabolic roles of any other taxa detected in this study. 

In terms of corrosion risk, the nitrate- and nitrite-treatment experiments, as performed here, showed low (<0.025 mm/year) to moderate (0.025–0.12 mm/year) corrosion rates, aside from a few replicates that showed high (0.13–0.25 mm/year) to severe (>0.25 mm/year) general corrosion rates (Figure 6) [27]. Varying pitting frequency was also observed (Figure 6). To attribute cause to the corrosion rates and pitting frequency observed in this study is difficult, given the use of environmental samples harboring diverse microbial communities and metabolisms. Further, in many cases in this study, there was poor consistency between the sampling locations and treatments, with regard to the general corrosion rates and pitting counts, making correlations to specific microbial community members challenging. However, the central conclusions from the data obtained point toward the no-additive (untreated) condition as the highest risk for MIC, and the S_nitrite treatments as the highest risk for chemical corrosion. Thus, nitrate or nitrate treatments as applied in these thermophilic microcosm experiments using topside fluids did not exacerbate corrosion rates due to microbial activity.

Some previous laboratory-based studies testing the effects of nitrate or nitrate on souring control and/or on MIC potential showed overall higher general corrosion rates [9,10,13], than we found here. Exogenous, labile carbon sources were added in some of these previous studies and/or they were conducted at mesophilic temperatures and under flowing conditions. In contrast, in the present study, no exogenous organic carbon or energy source was added (aside from the carbon steel coupons, which can serve as an energy source [2,6]), and thermophilic conditions were used. Further, treatments were only performed at the start of the 156-day batch experiment. Such experimental differences, along with the microbial communities associated with the specific FPSO topside machinery sampled here, presumably account for the differences in the reported corrosion rates in the presence of nitrate or nitrite. Despite these differences, it is valuable to perform studies such as this to build data sets that document how field microbial communities respond to various chemistries that would result from souring treatments under different conditions, in order to better predict their success and circumvent detrimental impacts, such as corrosion, when used in the industry. 

Overall, this study identified (1) the similarities and differences of microbial communities between the sampling locations on FPSO platforms drawing from oil reservoirs located in the same geographical basin, (2) that topside-produced water with no nitrogen or sulfur species additives had the highest risk of sulfate reduction and suffered the highest general corrosion rates, and, finally, (3) that nitrite treatments were effective in long-term sulfate reduction inhibition in samples, but conferred chemical corrosion risks. Overall, the results provide a description of nitrogen- and sulfur-based microbial activities, and their risk for MIC that can occur along fluid processing lines on FPSO topsides. Additional studies that use produced water fluids associated with offshore topside infrastructure to test the effects of nitrate and nitrite on souring and MIC under thermophilic conditions will help to augment our findings.

## Figures and Tables

**Table 1:** Characteristics of the produced water samples collected from each location on the topsides of Platforms A and B.

Sample	Platform	Log No. Cells per mL ^a^	Salinity (M Eq. NaCl)	Temp at Sampling (°C)	pH	S^2−^ (mM)	SO_4_^2−^ (mM)	Total Fe^2+/3+^ (mM)	NH_4_^+^ (mM)	Acetate (mM)
A_MPSep	A	4	0.54	54	7.5	0	9.3	0	1.27	3.22
A_MPHC	A	5	0.55	54	7.9	0	8.6	0	1.43	3.18
A_TestHC	A	5	0.57	55	7.9	0	8.1	0	1.41	2.02
B_MPHC	B	6	0.51	60	7.1	0	22.0	0.53	1.20	0.79
B_TestSep	B	6	0.47	60	7.0	0	19.4	0.60	1.38	1.02
B_TestHC	B	6	0.49	54	7.1	0	23.8	0.53	1.59	1.13

^a^ Log number of microbial cells per mL determined by the ATP assay.

**Table 2:** Nitrite accumulation (mM) in nitrate-amended Platform B microcosms after a 156-day incubation period.

Sample	Treatment	Nitrite Production (mM)		
**B_MPHC**	Nitrate	0.78	0	1.03
	S_nitrate	0.96	1.11	0
	Control	0	0	0
**B_TestSep**	Nitrate	0.29	2.10	2.02
	S_nitrate	0	1.78	2.45
	Control	0	0	0
**B_TestHC**	Nitrate	0	0	0
	S_nitrate	1.87	0.37	0
	Control	0	0	0

## Data Availability

The data presented in this study are available within the article.
